# Supplementary material for: Multimorbidity and health-related quality of life among patients attending chronic outpatient medical care in Bahir Dar, Northwest Ethiopia: The application of partial proportional odds model
Source: PLOS Glob Public Health. 2022 Nov 2;2(11):e0001176. doi: 10.1371/journal.pgph.0001176 (PMC10021695; doi:10.1371/journal.pgph.0001176)
Supplement: S1 File — (PDF) [file pgph.0001176.s003.pdf]

SF-12® Patient Questionnaire

Patient Initials _____ _____ ______ **Date of Birth:** ___/___/___ **Patkey:** ______

Surgeon Name: ____ **Date:** ____________

Examination Period:
_____ Preop (1)
_____ Immediate Postop (2)
_____ 1 Year (3)
_____ 3 Year (4)
_____ 5 Year (5)
_____ Other (specify) (6): _____________

SF-12®:
This information will help your doctors keep track of how you feel and how well you are able to do your usual activities. Answer every question by placing a check mark on the line in front of the appropriate answer. It is not specific for arthritis. If you are unsure about how to answer a question, please give the best answer you can and make a written comment beside your answer.

1. In general, would you say your health is:
   _____ Excellent (1)
   _____ Very Good (2)
   _____ Good (3)
   _____ Fair (4)
   _____ Poor (5)

The following two questions are about activities you might do during a typical day. Does YOUR HEALTH NOW LIMIT YOU in these activities? If so, how much?

2. MODERATE ACTIVITIES, such as moving a table, pushing a vacuum cleaner, bowling, or playing golf:
   _____ Yes, Limited A Lot (1)
   _____ Yes, Limited A Little (2)
   _____ No, Not Limited At All (3)

3. Climbing SEVERAL flights of stairs:
   _____ Yes, Limited A Lot (1)
   _____ Yes, Limited A Little (2)
   _____ No, Not Limited At All (3)

During the PAST 4 WEEKS have you had any of the following problems with your work or other regular activities AS A RESULT OF YOUR PHYSICAL HEALTH?

4. ACCOMPLISHED LESS than you would like:
   _____ Yes (1)
   _____ No (2)

5. Were limited in the KIND of work or other activities:
   _____ Yes (1)
   _____ No (2)

Surgeon Initials __________ **Date:** _____________

Patient Initials _____ _____ ______ **Date of Birth:** ___/___/___ **Patkey:** ______

Surgeon Name: ____ **Date:** ____________

Examination Period: _____ Preop (1)
_____ Immediate Postop (2)
_____ 1 Year (3)
_____ 3 Year (4)
_____ 5 Year (5)
_____ Other (specify) (6): ____________

SF-12® Cont'd:

During the PAST 4 WEEKS, were you limited in the kind of work you do or other regular activities AS A RESULT OF ANY EMOTIONAL PROBLEMS (such as feeling depressed or anxious)?

6. ACCOMPLISHED LESS than you would like:
   _____ Yes (1)
   _____ No (2)

7. Didn't do work or other activities as CAREFULLY as usual:
   _____ Yes (1)
   _____ No (2)

8. During the PAST 4 WEEKS, how much did PAIN interfere with your normal work (including both work outside the home and housework)?
   _____ Not At All (1)
   _____ A Little Bit (2)
   _____ Moderately (3)
   _____ Quite A Bit (4)
   _____ Extremely (5)

The next three questions are about how you feel and how things have been DURING THE PAST 4 WEEKS. For each question, please give the one answer that comes closest to the way you have been feeling. How much of the time during the PAST 4 WEEKS –

9. Have you felt calm and peaceful?
   _____ All of the Time (1)
   _____ Most of the Time (2)
   _____ A Good Bit of the Time (3)
   _____ Some of the Time (4)
   _____ A Little of the Time (5)
   _____ None of the Time (6)

Surgeon Initials __________ **Date:** _____________

Patient Initials _____ _____ ______ **Date of Birth:** ___/___/___ **Patkey:** ______

Surgeon Name: ____ **Date:** ____________

Examination Period:
_____ Preop (1)
_____ Immediate Postop (2)
_____ 1 Year (3)
_____ 3 Year (4)
_____ 5 Year (5)
_____ Other (specify) (6): ____________

SF-12® Cont'd:

10. Did you have a lot of energy?
_____ All of the Time (1)
_____ Most of the Time (2)
_____ A Good Bit of the Time (3)
_____ Some of the Time (4)
_____ A Little of the Time (5)
_____ None of the Time (6)

11. Have you felt downhearted and blue?
_____ All of the Time (1)
_____ Most of the Time (2)
_____ A Good Bit of the Time (3)
_____ Some of the Time (4)
_____ A Little of the Time (5)
_____ None of the Time (6)

12. During the PAST 4 WEEKS, how much of the time has your PHYSICAL HEALTH OR EMOTIONAL PROBLEMS interfered with your social activities (like visiting with friends, relatives, etc.)?
_____ All of the Time (1)
_____ Most of the Time (2)
_____ A Good Bit of the Time (3)
_____ Some of the Time (4)
_____ A Little of the Time (5)
_____ None of the Time (6)

Surgeon Signature____ **Date**__________________________

SF-12® Health Survey © 1994, 2002 by Medical Outcomes Trust and QualityMetric Incorporated. All Rights Reserved
SF-12® is a registered trademark of Medical Outcomes Trust
