# Supplementary material for: Multimorbidity and health-related quality of life among patients attending chronic outpatient medical care in Bahir Dar, Northwest Ethiopia: The application of partial proportional odds model
Source: PLOS Glob Public Health. 2022 Nov 2;2(11):e0001176. doi: 10.1371/journal.pgph.0001176 (PMC10021695; doi:10.1371/journal.pgph.0001176)
Supplement: Attachment — Submitted filename: Responses to Reviewers.docx [file pgph.0001176.s004.docx]

**Point-by-point responses to editor’s and reviewers’ comments**

PGPH-D-22-01200
Multimorbidity and health-related quality of life among patients attending chronic outpatient medical care in Bahir Dar, Northwest Ethiopia: the application of partial proportional odds model
PLOS Global Public Health

Dear Dr. Eyowas,

Thank you for submitting your manuscript to PLOS Global Public Health. After careful consideration, we feel that it has merit but does not fully meet PLOS Global Public Health’s publication criteria as it currently stands. Therefore, we invite you to submit a revised version of the manuscript that addresses the points raised during the review process.

Authors’ response:

Thank you so much for the opportunity given to us to revise and submit the manuscript. The comments and suggestions given are valuable and educational. We have addressed all the comments accordingly and indicated the line and page numbers of the changes made in the manuscript body.

==============================
EDITOR: Please address the critical comments of the two reviewers. It's important that you re-check for any grammatical and punctuation problems throughout the manuscript.

Authors’ response:

Thank you for the suggestions. The authors re-checked the manuscript for grammatical and punctuation errors and proofread it accordingly.
==============================

Please submit your revised manuscript by Sep 28 2022 11:59PM. If you will need more time than this to complete your revisions, please reply to this message or contact the journal office at [globalpubhealth@plos.org](mailto:globalpubhealth@plos.org). When you're ready to submit your revision, log on to <https://www.editorialmanager.com/pgph/> and select the 'Submissions Needing Revision' folder to locate your manuscript file.

Please include the following items when submitting your revised manuscript:

- A rebuttal letter that responds to each point raised by the editor and reviewer(s). You should upload this letter as a separate file labeled 'Response to Reviewers'.
- A marked-up copy of your manuscript that highlights changes made to the original version. You should upload this as a separate file labeled 'Revised Manuscript with Track Changes'.
- An unmarked version of your revised paper without tracked changes. You should upload this as a separate file labeled 'Manuscript'.

Authors’ response:

We have included all the items required during resubmission.

Guidelines for resubmitting your figure files are available below the reviewer comments at the end of this letter.

We look forward to receiving your revised manuscript.

Authors’ response:

Thank you for the guidance and motivation.

Journal Requirements:

1. Please update your online Competing Interests statement. If you have no competing interests to declare, please state: “The authors have declared that no competing interests exist.”

Authors’ response:

Thank you. We have updated the statement both online and on the revised manuscript. Page 33, line 605.

2. Please amend your online detailed Financial Disclosure statement. This is published with the article. It must therefore be completed in full sentences and contain the exact wording you wish to be published.

a. State the initials, alongside each funding source, of each author to receive each grant.
   b. State what role the funders took in the study. If the funders had no role in your study, please state: “The funders had no role in study design, data collection and analysis, decision to publish, or preparation of the manuscript.”

Authors’ response:

Thank you. The funding statement is revised as suggested. Page 32, line 588.

3. Please provide a complete Data Availability Statement in the submission form, ensuring you include all necessary access information or a reason for why you are unable to make your data freely accessible. If your research concerns only data provided within your submission, please write “All data are in the manuscript and/or supporting information files.” as your Data Availability Statement.

Authors’ response:

Thank you. The data availability statement is revised as commented. Page 33, line 605

4. Please include a title page at the beginning of your manuscript file, that lists full author names and institute addresses. Do not upload as a separate file.

Authors’ response:

Thank you for the advice. We have included a title page at the beginning of the manuscript file and removed the one submitted as a separate file. Page 1. We have checked the full names and institutional addresses

5. Your manuscript is missing the following sections: Abstract. Please ensure these are present, and in the correct order, and that any references to subheadings in your main text are correct. An outline of the required sections can be consulted in our submission guidelines here: <https://journals.plos.org/globalpublichealth/s/submission-guidelines#loc-parts-of-a-submission>

Authors’ response:

Thank you for the comments. We have placed the abstract next to the title page in the manuscript file, Page 2. We also checked that the references in the main text are correct.

6. Please provide separate figure files in .tif or .eps format only and ensure that all files are under our size limit of 10MB.

For more information about how to convert your figure files please see our guidelines: <https://journals.plos.org/globalpublichealth/s/figures>

Authors’ response:

Thank you. We submitted all the figure files in .tif format.

7. We have noticed that you have uploaded Supporting Information files, but you have not included a list of legends. Please add a full list of legends for your Supporting Information files after the references list.

Authors’ response:

Thank you. A full list of titles are written for all the supporting information submitted after the reference list. Page 41.

8. Please review your reference list to ensure that it is complete and correct. If you have cited papers that have been retracted, please include the rationale for doing so in the manuscript text, or remove these references and replace them with relevant current references. Any changes to the reference list should be mentioned in the rebuttal letter that accompanies your revised manuscript. If you need to cite a retracted article, indicate the article’s retracted status in the References list and also include a citation and full reference for the retraction notice.

 Authors’ response:

Thank you. We have checked the reference list for completeness and correctness. We have also checked the in-text citations. We revised reference # 19 and 61. We could not identify that any retracted papers have been cited in our manuscript (we checked them using scite_ software). We have added some references based on reviewers suggestions and to discuss the findings and changes in the results following the additional data analysis techniques suggested by the reviewers.

Review Comments to the Author

Reviewer #1: This is a well written article. The study is well-conceived (I read your published protocol) and very worthwhile.
I liked everything very much except that I would like you to please consider redoing the univariate and multivariate regression. These should be done using Stata's survey module. As you know, I am sure, first svyset. Then svy: for the univariate and gsvy: for the gologit2 command.
Authors responses:

Thank you very much for the recommendation to redo the analyses considering sampling stratification using svyset (weighting), svy and gsvy prefixes in ologit and gologit2 commands, respectively. We have redone the analyses using the suggested commands and reported the results accordingly. Indeed, there were changes in the parameter estimate and level of significance when weighting is applied both in the univariate and multivariate analysis models. Based on the new results, we have revised the methodology, results, discussion, recommendation and the abstract as well. The comments given helped to substantially improve the quality of the manuscript. The revisions made are indicated from page 12-14, 20-26 and from 27-29.

I also feel that the structure of the sampling should be a bit clearer in the methods section. At the moment you mention stratification but the strata appear to be more than two and this look more like PSUs to me; whereas, after reading your protocol it is clear there were two strata; public and private sectors. Then there was two stage random and systematic sampling within these two strata. Normally one tries to meet the sample size for each stratum. In your case you allocated numbers pro rata to each based on historical utilisation. This may make the weighting easier to deal with. Nevertheless the two Strata should be identified as well as the clusters within each stratum. It would also be best practice to publish a table of design effects so that other researchers can use these when deciding on their own sample sizes.

Authors responses:

Thank you again for recommending to clarify this section. As you said, we employed a stratified sampling method. The stratification was made based the nature of health facilities involved in the study (public and private). The sample size for each facility was determined based on the total sample size required for the entire study and the volume of patients registered for follow up over the year preceding the survey in each eligible facility. As we did a random sampling of patients from each facility, the notion of clustering was not that much needed. We have considered a design effect of 2 to allow maximum variation and for a plausible sub-group analysis. Fortunately, the DEFF value estimated by the svy: ologit and gsvy:gologit2 was equal to our allocation (Design df = 2). We do appreciate that comment.

Reviewer #2: This is a great study that adds to the NCD literature of multimorbidity in LMIC. Data was collected through two different sources which strengthens the study data quality.

Authors’ response:

Thank you for the positive feedback.
Introduction:
The introduction is very thorough and complete however it would benefit from additional reviews or studies done in LMIC or regional/local (Ethiopia) studies for multimorbidity:
• Bhagavathula AS,. Prevalence and Determinants of Multimorbidity, Polypharmacy, and Potentially Inappropriate Medication Use in the Older Outpatients: Findings from EuroAgeism H2020 ESR7 Project in Ethiopia. Pharmaceuticals (Basel). 2021 Aug 25;14(9):844. doi: 10.3390/ph14090844. PMID: 34577544; PMCID: PMC8468438.
• Abebe F, Schneider M, Asrat B, Ambaw F. Multimorbidity of chronic non-communicable diseases in low- and middle-income countries: A scoping review. J Comorb. 2020 Oct 16;10:2235042X20961919. doi: 10.1177/2235042X20961919. PMID: 33117722; PMCID: PMC7573723.

Authors’ response:

Thank you for the recommendations. We have used the articles to strengthen the background section. The second article was authored by the authors of this manuscript and was already used in the background section (#67 in the previous version and #6 in the current version).

Methods:
The study methodology has been clearly described.
The authors mention the use of SF-12 as a data collection instrument. Authors should consider to add the SF-12 tool as an appendix

Authors’ response:

Thank you for the suggestion. We have uploaded the tool (which is publicly available) along with other supplemental files.

Results
Line 236 Lifestyle and psychosocial characteristics: It is confusing that the authors talk about BMI and include Figure 1, but in the same paragraph they talk about social support – which there is no graph or table for it. If left as is I would recommend splitting the paragraph and adding a note saying (data not shown in tables)

Authors’ response:

Thank you for the comments. We have split the paragraph and described the results separately. We have also shown the figure next to the BMI description. The description referring to social support is given separately and we indicated that no data were shown for the psychosocial support system in the text. Page 16, line 333-341.

Line 275 -314 would fit better under methods rather than results

Authors’ response:

Thank you for the suggestion. We have taken the text referring to methods in the methodology section. Page 12, line 273 to page 14, line 316.

Discussion
It would be interesting to include risk factors into the study as well (tobacco use, physical inactivity, obesity, alcohol use) – this could be added as a discussion point. Freisling H, et,.al. Lifestyle factors and risk of multimorbidity of cancer and cardiometabolic diseases: a multinational cohort study. BMC Med. 2020 Jan 10;18(1):5. doi: 10.1186/s12916-019-1474-7. PMID: 31918762; PMCID: PMC6953215.

Authors’ response:

Thank you for the recommendations. As in the background section, we discussed the common risk factors of multimorbidity in the discussion section. We have cited the proposed article both in the background and discussion section. In the discussion, we described this as follows “Studies show the most common risk factors contributing for the increasing burden of multimorbidity are advanced age, obesity, physical inactivity, socioeconomic deprivation and use of tobacco and alcohol.” Page 27, line 480-484

The following statement made earlier in the discussion should go under study strengths and limitations:
The authors used the very commonly used QoL measure, the SF-12V2 362 tool. However, the method of analysis we employed- the partial proportional odds (PPO) is relatively new 22 363 in the context of analyzing QoL data(77). The PPO model is said to be a robust QoL data analytic method 364 compared to other methods of analysis provided the nature of a given data warrants its use(41).

Authors’ response:

Thank you for the recommendations. We have taken these statements to the study strengths and limitations section. Page 32, line 590-594
Table 2
Superscript a mentions the definition of all other NCDs which the footnote specifies to be asthma, COPD, stroke, cancer and depression. However in the methods (and in

Figure 2) the authors mention many more NCDs not mentioned in Table 2. Please clarify for the reader if these other chronic conditions were taken out of the analysis (degenerative nerve diseases, thyroid diseases, gastrointestinal diseases)

Authors’ response:

Thank you for the comments. Other NCDs in the table 2 refer to the less prevalent NCDs. We collapsed the lists into “Other” to minimize the length of the table. As you advised, we have given a footnote under the table to clarify “others”. Page 19, line 362.

Figures:
Figure 1- strongly recommend not using pie charts for visualizing more than 2 groups: The issue with pie chart ([data-to-viz.com](http://data-to-viz.com/))

Authors’ response:

Thank you for the feedback. We changed the pie chart and presented the data by bar graph. Figure 1 (sup-files, figures)
Figures already have at the bottom the legend and description so recommend deleting the top title to avoid confusion (for example: Figure 2 Common NCDs identified Vs List of NCDS studied and their magnitude …)

Authors’ response:

Thank you. We have revised and indicated on the bottom description.
Please add what the numbers in the graphs represent (percentages, N, etc)

Authors’ response:

Thank you for the recommendations. We clarified what the numbers represent in each figure.
Figure 5 – check for a mistake/typo in “severe”.

Authors’ response:

Thank you. We revised the typo error.

[NOTE: If reviewer comments were submitted as an attachment file, they will be attached to this email and accessible via the submission site. Please log into your account, locate the manuscript record, and check for the action link "View Attachments". If this link does not appear, there are no attachment files.]

While revising your submission, please upload your figure files to the Preflight Analysis and Conversion Engine (PACE) digital diagnostic tool, <https://pacev2.apexcovantage.com/>. PACE helps ensure that figures meet PLOS requirements. To use PACE, you must first register as a user. Registration is free. Then, login and navigate to the UPLOAD tab, where you will find detailed instructions on how to use the tool. If you encounter any issues or have any questions when using PACE, please email PLOS at [figures@plos.org](mailto:figures@plos.org). Please note that Supporting Information files do not need this step.

Authors Response

Thank you. We have used the PACE tool to covert the and uploaded them accordingly.
