# Supplementary material for: Multimorbidity and health-related quality of life among patients attending chronic outpatient medical care in Bahir Dar, Northwest Ethiopia: The application of partial proportional odds model
Source: PLOS Glob Public Health. 2022 Nov 2;2(11):e0001176. doi: 10.1371/journal.pgph.0001176 (PMC10021695; doi:10.1371/journal.pgph.0001176)
Supplement: Attachment — Submitted filename: Point-by-point responses for editor comment re2.docx [file pgph.0001176.s005.docx]

Point-by-point response to the editor comment

Thanks for addressing the comments raised by the reviewer. The paper would benefit from the use of an appropriate multimorbidity index (see:[https://www.bmj.com/content/368/bmj.m160](https://www.bmj.com/content/368/bmj.m160))

Authors response:

Thank you for the suggestion!

Multimorbidity can be measured from different perspectives (depending on the aim of the study and availability of data). For the sake of comparison with other literature and feasibility, we applied conventional methods, perhaps by combining different sources of data (self-report + review of medical records for physical morbidity and direct assessment of mental health condition (depression).

The aim of this study was to assess the impact of multimorbidity on QoL (as an important outcome measure/index). We used the SF-12 QoL assessment tool, which is validated to measure QoL in the context of multimorbidity studies.

As we read from the article that we were advised to refer, the method we applied is one of the variety of ways to measure and we believe it is plausible and that it is sufficient for the objectives we had.

Thank you,

Fantu Abebe
